# Prediction of Sustained Virological Response to Peginterferon-based Therapy for Chronic Hepatitis C: Regression Analysis of a Cohort from Rio Grande do Sul, Brazil

**DOI:** 10.5005/jp-journals-10018-1207

**Published:** 2017-05-05

**Authors:** Rafael V Picon, Lúcia Fendt, Karine Amaral, Paulo D Picon

**Affiliations:** 1 Department of Gastroenterology, Hospital Nossa Senhora da Conceição, Porto Alegre, Rio Grande do Sul, Brazil; 2 Department of Critical Care, Hospital de Clínicas de Porto Alegre, Porto Alegre, Rio Grande do Sul, Brazil; 3 Department of Exceptional Drug Access Committee, Hospital de Clinicas de Porto Alegre, Porto Alegre, Rio Grande do Sul, Brazil; 4 Department of Internal Medicine, Hospital de Clinicas de Porto Alegre, Porto Alegre, Rio Grande do Sul, Brazil

**Keywords:** Cohort study, Hepatitis C virus, Logistic regression, Peginterferon, Therapy.

## Abstract

**Aim:**

Peginterferon plus ribavirin (peg-IFN/RBV) is still the standard of care for treatment of hepatitis C virus (HCV) in many countries. Given the high toxicity of this regimen, our study aimed to develop a prediction tool that can identify which patients are unlikely to benefit from peg-IFN/RBV and could thus postpone treatment in favor of new-generation direct-acting antivirals.

**Materials and methods:**

Binary regression was performed using demographic, clinical, and laboratory covariates and sustained virological response (SVR) outcomes from a prospective cohort of individuals referred for therapy from 2003 to 2008 in a public HCV treatment center in Rio Grande do Sul, Brazil.

**Results:**

Of the 743 participants analyzed, 489 completed 48 weeks of treatment (65.8%). A total of 202 of those who completed peg-IFN/RBV therapy achieved SVR (27.2% responders), 196 did not (26.4%), and 91 had missing viral load (VL) at week 72 (12.2% loss to follow-up). The remainder discontinued therapy (n = 254 [34.2%]), 78 (30.7%) doing so due to adverse effects. Baseline covariates included in the regression model were sex, age, human immunodeficiency virus, infection status, aspartate transaminase, alanine transaminase, hemoglobin, platelets, serum creatinine, prothrombin time, pretreatment VL, cirrhosis on liver biopsy, and treatment naivety. A predicted SVR of 17.9% had 90.0% sensitivity for detecting true nonresponders. The negative likelihood ratio at a predicted SVR of 17.9% was 0.16, and the negative predictive value was 92.6%.

**Conclusion:**

Easily obtainable variables can identify patients that will likely not benefit from peg-IFN-based therapy. This prediction model might be useful to clinicians.

**Clinical significance:**

To our knowledge, this is the only prediction tool that can reliably help clinicians to postpone peg-IFN/RBV therapy for HCV genotype 1 patients.

**How to cite this article:** Picon RV, Fendt L, Amaral K, Picon PD. Prediction of Sustained Virological Response to Peginterferon-based Therapy for Chronic Hepatitis C: Regression Analysis of a Cohort from Rio Grande do Sul, Brazil. Euroasian J Hepato-Gastroenterol 2017;7(1):27-33.

## INTRODUCTION

Hepatitis C virus (HCV) infection has an estimated worldwide prevalence of 2.35%.^1^ In Brazil, population-based studies show moderate prevalence rates of HCV infection ranging from 0.7 to 2.1%.^2^ The natural history of the disease is extremely variable: Most hosts remain asymptomatic throughout their entire lives and often go undiagnosed for long periods; others progress to liver cirrhosis and hepatocellular carcinoma (HCC) over the course of many years to decades,^3^ with high rates of complications and substantial morbidity.

A combination of peginterferon (peg-IFN) alfa-2a or alfa-2b and ribavirin (RBV) is the standard of care for HCV treatment and, in many countries, is still the only therapeutic option available other than conventional IFN.

It is still the standard therapy in Egypt, for instance, a country with extremely high prevalence of HCV.^4^ As of 2013, newer drugs became available in Brazil through private purchasing and within the publicly funded Unified Health System (Sistema Único de Saúde, SUS): The direct-acting antivirals (DAA) boceprevir and tela-previr,^5^ both in the protease inhibitor class. Later in 2013, the US Food and Drug Administration approved two even newer DAAs, the first of a new generation of drugs: Simeprevir and sofosbuvir.^6^ Both received marketing approval from the Brazilian National Health Surveillance Agency in 2015, as did another DAA, daclatasvir. These three drugs were added to the official Ministry of Health guidelines in June 2015.^7^

Until 2011, the only standard treatment regimen for HCV genotype 1 consisted of peg-IFN/RBV for 48 weeks, which yields sustained virological response (SVR) rates of 40 to 50% in randomized clinical trials (RCTs).^8^ The SVR rates achieved with peg-IFN/RBV are themselves affected by a number of different variables: HCV genotype (non-1 genotypes are associated with higher SVR), pretreatment viral load (VL), degrees of fibrosis and inflammatory activity at liver biopsy, and host IL28B genotype (the CC genotype is associated with higher SVR).^9-11^

The intended purpose of treatment is to eradicate the virus and slow the progression of liver damage, thus preventing HCV-related complications. However, the side effects of treatment are significant, leading to substantial morbidity and quality-of-life impairment. The cost of treatment also places a heavy burden on health systems. Due to the potential for complications and the low SVR rates achieved with standard therapy, the decision to start HCV treatment and the optimal timing of treatment during the course of the disease are subject to debate. The expected increase in availability of new-generation DAAs justifies particularly judicious prescription of peg-IFN/RBV dual therapy at the present time, as selected patients able to defer treatment may be candidates for new-generation DAA therapy in the future, with improved SVR rates.

Although several studies have postulated variables that may predict SVR to peg-IFN/RBV treatment, to date, no tool has been developed to assist in the decision to begin or postpone therapy. Hence, this study sought to mathematically determine which patients are least likely to respond to peg-IFN/RBV therapy. This information could then be used to develop a decision aid to assist in defining whether to defer peg-IFN/RBV treatment of individuals with genotype 1 HCV infection and formal indications for therapy, but in whom risk-benefit ratio is a particular concern.

## MATERIALS AND METHODS

### Study Design

*Post hoc* analysis of a prospective cohort study in a dynamic population.

### Patients

The data analyzed in this study were obtained from a prospective cohort of 752 patients with HCV infection referred to receive peg-IFN/RBV therapy at a hospital-based statewide referral center in Southern Brazil. Enrollment took place between September 2003 and March 2008.

All patients referred for treatment and included in the cohort met the eligibility criteria for peg-IFN therapy set out in the relevant Ministry of Health Clinical Protocol and Therapeutic Guideline: METAVIR any A and ≥F2, or ≥A2 and ≥F1.^12^ Only those patients referred for a 48-week therapy regimen were included in the regression model.

### Treatment

The peg-IFN/RBV therapy was provided at standard doses. Two types of peg-IFN were used and compared: peg-IFN alfa-2a at a dose of 180 μg/kg and peg-IFN alfa-2b at a dose of 1.5 ug/kg, both administered once weekly. The RBV was administered at a dose of 1,250 mg/day to patients weighing > 75 kg or 1,000 mg/day to those weighing ≤75 kg.

At baseline, clinical data (sex, age, comorbidities, prior treatment) and laboratory parameters [bilirubin, prothrombin time (PT), albumin, glucose, uric acid, transaminases, complete blood count, platelet count, creatinine, thyroid-stimulating hormone, quantitative polymerase chain reaction, and biopsy] and during follow-up, adverse effects were monitored through periodic interviews by a pharmacist and monthly laboratory tests.

### Outcome Measure

Quantitative VL testing was performed on treatment week 12 for characterization of early virological response, defined as an undetectable VL or a 2 × log_10_ IU/mL or greater drop in VL. Nonresponders had treatment discontinued as per protocol. Qualitative VL testing was performed on week 52 for assessment of virological response (planned treatment completion) and on week 72 (i.e., 24 weeks after treatment completion) for characterization of SVR.

### Variable Selection and Statistical Analysis

A binary logistic regression model was constructed. The dependent variable was SVR, as defined above. This variable was selected as the best possible surrogate outcome, as we did not have access to clinical endpoint data (e.g., hospitalizations, HCC, liver transplantation, death, etc.). Some of the predictor variables were established based on bivariate analysis followed by multivariate analysis with Poisson regression, previously performed on this sample as part of an unpublished study. These variables were HIV coinfec-tion, presence of cirrhosis, age, and alanine transaminase (ALT). Therefore, all models developed for potential SVR prediction included at least some of these characteristics as independent variables. For the purposes of this study, cirrhosis was defined as the presence of grade IV fibrosis on liver biopsy according to the METAVIR classification (METAVIR F4).^13^ The other predictor variables, employed in various combinations in 22 distinct models, were aspartate transaminase (AST) in IU/L, hemoglobin (Hb) in g/dL, serum creatinine (Cr) in mg/dL, PT in seconds, serum albumin in g/dL, total bilirubin (Bt) in mg/dL, platelet count (PLT), VL in IU/mL, and treatment naivety.

Parameter estimation was performed by the maximum likelihood ratio method. Goodness of fit was evaluated using the Hosmer-Lemeshow test, with the significance level set at a = 0.05. The classification and predictive performance of each of the most promising models were evaluated by means of sensitivity, specificity, area under the receiver operating characteristic curve (AUROC), positive and negative likelihood ratios, and positive (PPV) and negative (NPV) predictive values derived using Bayes’ theorem. Missing data were not imputed, and the impact of losses to follow-up and consequent selection bias were assessed by analysis of the distribution of baseline characteristics between the study sample and the analyzed sample.

### Optimal Model and Cutoff Point

The optimal model, as defined *a priori,* would be that with the highest sensitivity for nonresponse to treatment, SVR NPV, and AUROC, the largest number of participants, and, in the interest of simplicity and applicability, the smallest possible number of covariates. Such a model would ensure discriminant capacity (responders *vs* nonresponders) and predictive power for nonresponse, thus minimizing erroneous classification of responders as nonresponders (false negative).

The optimal cutoff point for predicted probability of SVR to classify participants as responders or non-responders would be the value that ensured excellent performance for identifying true responders without threatening SVR NPV. Arbitrarily, the minimum acceptable sensitivity for identification of nonresponders was defined as 90%, as long as it did not jeopardize NPV to the point of defeating the purpose of the model. A Microsoft Excel-based SVR calculator was created using the equations from the best regression model and is provided as Additional file 1.

**Flow Chart 1:**
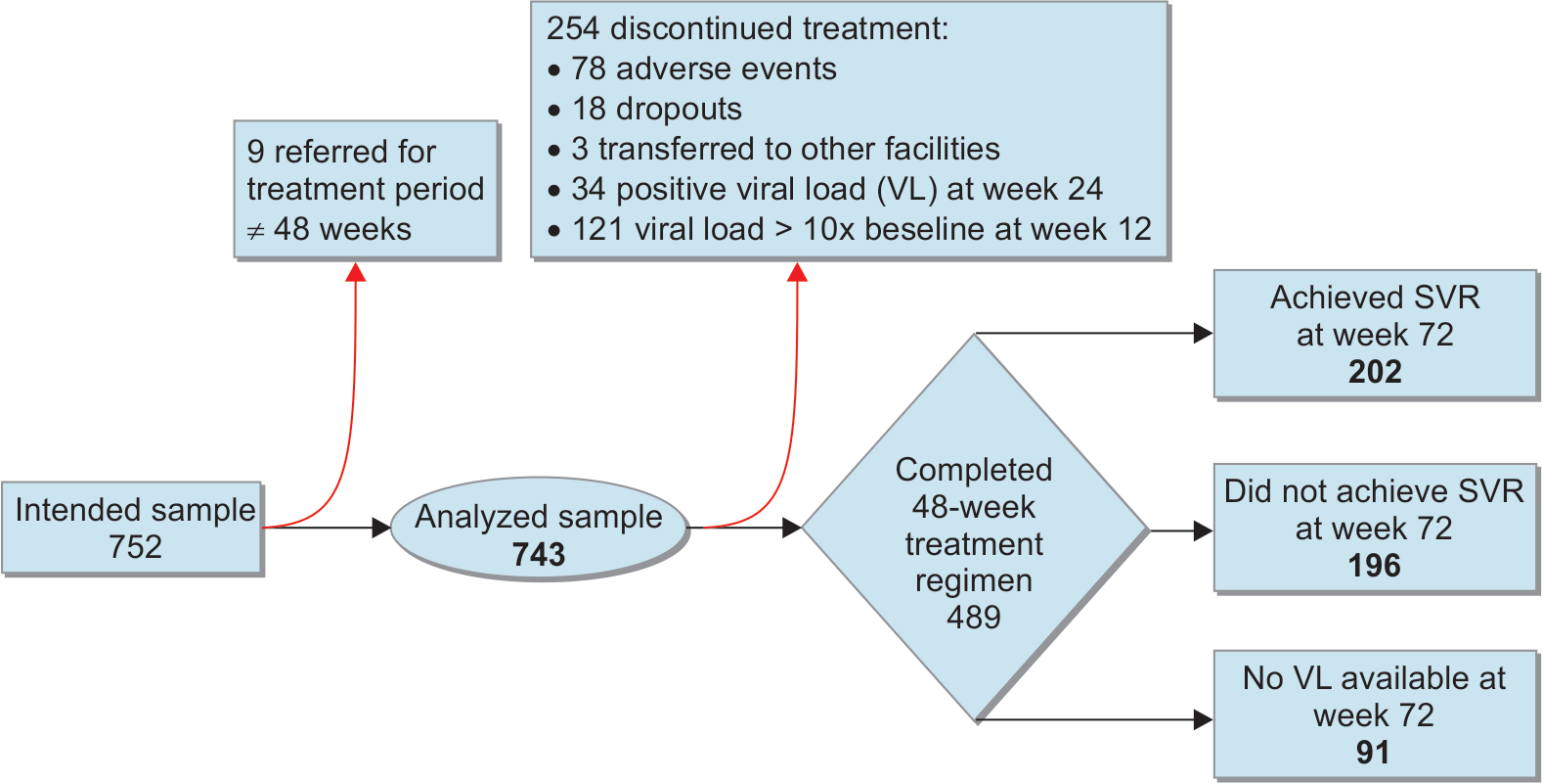
Participant flow diagram

### Ethical Aspects

This analysis of secondary data was approved by the Research Ethics Committee of our institution (opinion no. 032/06 of April 4, 2006). All patients enrolled in the original study provided written informed consent for participation.

## RESULTS

Flow Chart 1 shows a cohort participant flow diagram. Of the 752 participants referred for treatment, 9 were referred for regimens other than 48-week therapy and were, thus, excluded from analysis. Of the 743 participants analyzed, 489 (65.8%) completed the 48-week treatment period; 202 (27.2%) achieved SVR, and 196 (26.4%) did not. The remainder either discontinued treatment (n = 254, 34.2%) or had missing VL measurements for week 72 (n = 91, 12.2%).

Overall, 78 participants (10.5%) discontinued treatment due to adverse events, particularly decompen-sated cirrhosis and hematological abnormalities (data not shown), and 18 (2.4%) were lost to follow-up due to personal reasons. Nine patients died during treatment, three due to complications of decompensated cirrhosis (data not shown).

Table 1 describes the baseline characteristics of the studied sample. There was a narrow male predominance. The mean age was 50 years; only 10% of participants were being referred for retreatment, and 16.4% of participants were cirrhotic. Of 22 regression models developed, four were found to meet the defined prerequisites and were thus considered candidate models for comparative analysis. Table 2 lists the covariates of these four models (A, B, C, and D). Albumin was included as a variable only in model B and accounted for the major difference in number of participants analyzed in this model.

**Graph 1:**
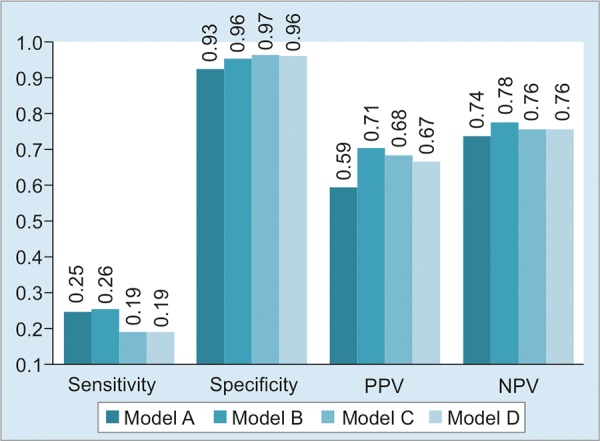
Performance of four candidate regression models

**Graph 2:**
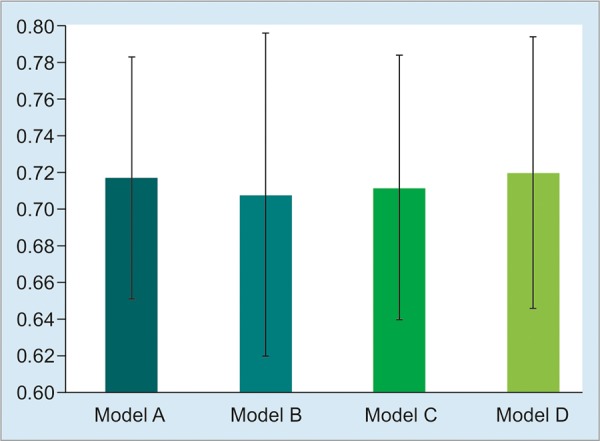
Classification performance of candidate models. Area under the ROC curve with 95% confidence intervals

Graph 1 illustrates the predictive performance of the candidate models. Specificity and NPV were similar across all four models. All models exhibited low sensitivity for identification of responders. There was no significant difference in overall classification capacity (evaluated by the AUROC) among the candidate models, as shown in Graph 2. Therefore, the models were similar.

**Table 1:** **Table 1:** Baseline characteristics of the studied sample (n = 743)

*Variable (n analyzed)*		*n (%)/mean (±SD)*	
Male sex		422 (56.8%)	
Age at treatment onset, years (n = 727)		50.0 (±10.5)	
Treatment naive		670 (90.8%)	
HIV coinfection		82 (11.0%)	
Prior liver biopsy		646 (86.9%)	
METAVIR F4 (cirrhosis)		122 (16.4%)	
ALT, IU/L (n = 612)		108.5 (±89.0)	
AST, IU/L (n = 611)		82.7 (±56.8)	
Total bilirubin, mg/dL (n = 355)		1.0 (±0.8)	
Serum creatinine, mg/dL (n = 515)		0.9 (±0.6)	
Prothrombin time, s (n = 387)		15.0 (±9.3)	
Serum albumin, g/dL (n = 290)		4.2 (±0.5)	
Hemoglobin, g/dL (n = 618)		14.3 (±1.5)	
Platelet count, × 10^3^/mm^3^ (n = 605)		180.5 (±72.3)	
Viral load, × 10^6^ IU/mL (n = 687)		4.1 (±10.8)	

**Table 2:** **Table 2:** Covariates of the four candidate regression models

		*Covariate*																											
*Model (n)*		*Sex*		*Age*		*HIV*		*ALT*		*AST*		*Hb*		*Cr*		*PT*		*Albumin*		*Bt*		*PLT*		*VL*		*Cirrhosis*		*Treatment naive*	
A (n = 254)		X		X		X		X		X		X		X		X		–		–		X		X		X		X	
B (n = 174)		X		X		X		X		X		–		–		X		X		X		X		–		X		X	
C (n = 246)		–		X		X		–		–		–		X		–		–		X		X		–		X		–	
D (n = 234)		–		X		X		–		–		–		X		–		–		X		X		X		–		–	

Model A was considered most promising, as it computed the largest number of participants (n = 254), with characteristics very similar to those of the overall sample (n = 743), as shown in Table 3. A predicted SVR probability of 17.9% ensured the minimum sensitivity of 90% in identifying nonresponders, as defined *a priori.* Therefore, a predicted probability of 17.9% was defined as the cutoff point for classification of participants as nonresponders. Model A’s specificity, negative likelihood ratio, positive likelihood ratio, NPV, and PPV at a predicted probability of 17.9% were 63%, 0.16, 2.43, 92.6%, and 54.8% respectively.

**Graph 3:**
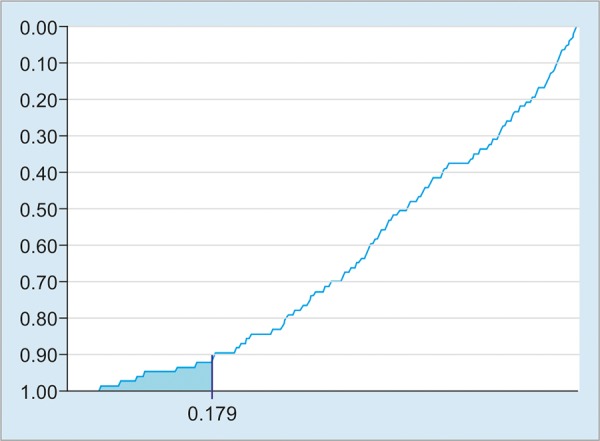
Sensitivity (reversed y-axis) as a function of SVR probability predicted by model A *(x*-axis). Shaded area: Proportion of responders misclassified as nonresponders at a 17.9% cutoff point

Graph 3 illustrates the small proportion of failures to detect responders (false negatives) with the 17.9% cutoff point. In the sample analyzed with model A, 31.5% of individuals had predicted SVR probability values <179% (data not shown).

**Table 3:** **Table 3:** Characteristics of the sample analyzed in model A *vs* the overall studied sample

		*%/mean (±SD)*			
*Variable*		*Model A*		*Study sample*	
Male sex		56.6%		56.8%	
Age at treatment onset, years (n = 727)		50.3 (±9.6)		50.0 (±10.5)	
Treatment naive		93.2%		90.8%	
HIV coinfection		9.6%		11.0%	
METAVIR F4 (cirrhosis)		17.1%		16.4%	
ALT, IU/L		104.14 (±87.2)		108.5 (±89.0)	
AST, IU/L		80.5 (±52.6)		82.7 (±56.8)	
Total bilirubin, mg/dL		1.0 (± 0.8)		1.0 (± 0.8)	
Serum creatinine, mg/dL		0.9 (±0.3)		0.9 (±0.6)	
Prothrombin time, s		15.0 (±9.2)		15.0 (±9.3)	
Serum albumin, g/dL		4.2 (±0.5)		4.2 (±0.5)	
Hemoglobin, g/dL		14.3 (±1.4)		14.3 (±1.5)	
Platelet count, × 10^3^/mm^3^		178.4 (±78.9)		180.5 (±72.3)	
Viral load, × 10^6^ IU/mL		4.2 (±12.3)		4.1 (±10.8)	

## EQUATIONS

The (y) function of binary logistic regression for model A is:

**Figure:**
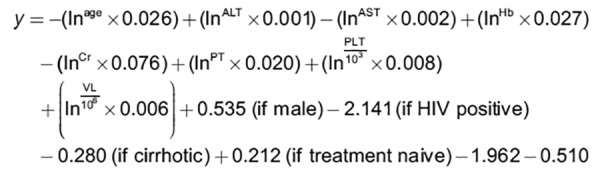


The probability of SVR (p_SVR_), considering a pretest probability of 33.3%, measured as a proportion ranging from 0 to 100%, was defined as:


*p_SVR_* = 100 ×(1- 0.333^e^y^^)


## DISCUSSION

The novelty of this study lies in its objective of developing a decision aid to assist in choosing whether to postpone peg-IFN/RBV therapy of HCV infection. Although several studies have assessed the influence of various factors on SVR, to the best of our knowledge, no research has sought to develop a predictive model for clinical use.

We believe the regression model described herein could be useful in clinical practice, as it is based on objective, easily obtainable variables, and can help refine indications for treatment beyond the recommendations of national clinical guidelines. The debate on optimal treatment timing is particularly relevant in light of the potentially severe adverse effects and high cost of peg-IFN/RBV dual therapy, and the introduction of more effective alternatives which are being slowly incorporated into practice. This model thus fits the concept of personalized medicine.

Model A proved particularly useful to support the decision to postpone treatment rather than to reinforce the decision to initiate treatment. This reflects the selected cutoff point of <17.9%, as we chose to prioritize superior NPV to the detriment of PPV. This decision was motivated by the greater clinical applicability of a model that could support treatment postponement while ensuring that treatment would not be denied to patients who could benefit from it. Simultaneously, we sought to establish a cutoff point that would encompass a substantial portion of the sample without failing to comply with the defined prerequisites. Using the cutoff point defined for model A, no more than 1 in 10 responders would be wrongly classified; approximately 3 out of 10 individuals referred for treatment would be classified by the model as non-responders eligible for treatment postponement; and at least 9 in every 10 patients classified as nonresponders would indeed fail to respond to therapy.

This cohort was composed of a group of patients referred to a SUS center for HCV treatment, which made the sample representative of routine clinical practice and ensured external validity within the publicly funded health system. Baseline characteristics and the proportion of treatment dropouts attributable to adverse events were consistent with those found in the literature. Although SVR rates were lower than those reported in randomized controlled trials,^8^ they were consistent with the findings of previous observational studies of effectiveness.^14,15^ This may be explained by the patient and comorbidity profiles, which could have been a reason for exclusion from RCTs.

The *post hoc* nature of our analysis is the most significant limitation of this investigation, as it may have concealed biases in the original study. However, as the predictor variables and outcome of interest were objective and easily determined, the impact of a potential measurement bias was mitigated. Losses attributable to missing data were significant, but internal validity was not compromised by selection bias, as the baseline characteristics of the subsample analyzed within model A were similar to those of the overall sample (Table 3). The high frequency of missing data is explained by the fact that pretreatment laboratory tests were selected and ordered at the discretion of the participants’ physicians rather than by the investigators.

The proposed model should be validated through *ad hoc* analyses. Future research may incorporate and use the findings presented herein to develop a specific model for prediction of SVR to novel anti-HCV therapies (i.e., new-generation DAAs). Until such a model is available and peg-IFN/RBV is entirely replaced by new alternatives, the findings of this study should be useful to support clinical decision-making worldwide.

## CLINICAL SIGNIFICANCE

To our knowledge, this is the only prediction tool that can reliably help clinicians to postpone peg-IFN/RBV therapy for HCV genotype 1 patients.

**Additional file 1:**
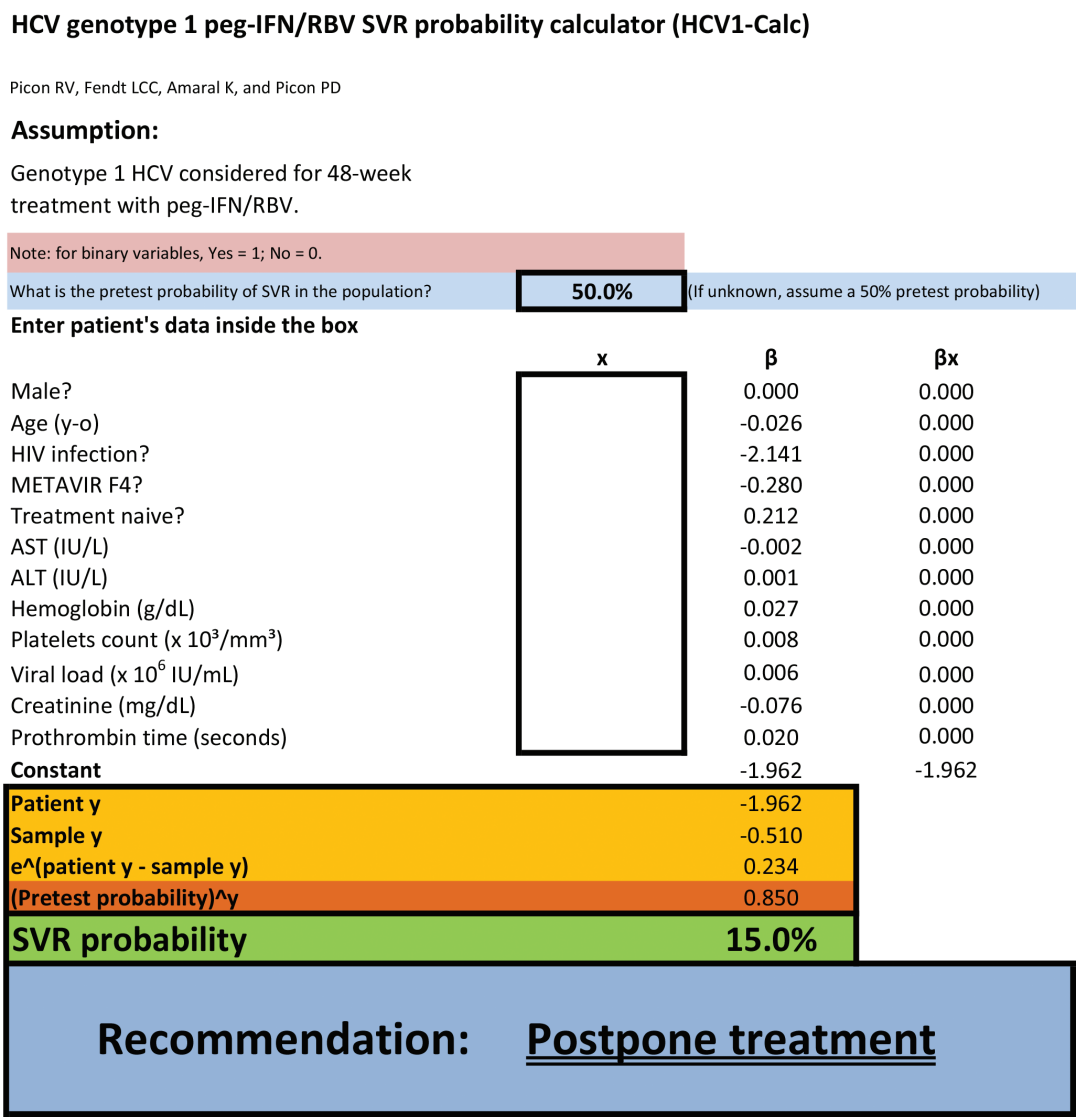
HCV genotype 1 peg-IFN/RBV SVR probability calculator (HCV1-Calc).
